# How do resource distribution and taxonomy affect the use of dual foraging in seabirds? A review

**DOI:** 10.1093/beheco/arad052

**Published:** 2023-07-11

**Authors:** Jessica A Phillips, Tim Guilford, Annette L Fayet

**Affiliations:** Department of Zoology, Oxford University, 11a Mansfield Rd, Oxford OX1 3SZ, UK; Ocean Networks Canada, 2474 Arbutus Road, Victoria, BC V8N 1V8, Canada; Department of Zoology, Oxford University, 11a Mansfield Rd, Oxford OX1 3SZ, UK; Department of Zoology, Oxford University, 11a Mansfield Rd, Oxford OX1 3SZ, UK; Norwegian Institute for Nature Research, Høgskoleringen 9, 7034 Trondheim, Norway

**Keywords:** central-place foraging, dual foraging, habitat quality, interspecific differences, parent–offspring conflict, seabirds

## Abstract

In many seabird species, parents feeding young switch between short and long foraging excursions in a strategy known as “dual foraging.” To investigate whether habitat quality near breeding colonies drives the use of dual foraging, we conducted a review of the seabird literature, compiling the results of 102 studies which identified dual-foraging in 50 species across nine families from all six seabird orders. We estimated the mean distance from the colony of each species’ short and long foraging trips and obtained remote-sensed data on chlorophyll-a concentrations within the radius of both short and long trips around each colony. We then assessed, for each seabird family, the relationship between the use of dual foraging strategies and the difference in the quality of foraging locations between short- and long-distance foraging trips. We found that the probability of dual foraging grew with increasing differences in the quality of foraging locations available during short- and long-distance trips. We also found that when controlling for differences in habitat quality, albatrosses and penguins were less likely to use dual foraging than Procellariidae, which in turn were less likely to use dual foraging than Sulids. This study helps clarify how environmental conditions and taxon-specific characteristics influence seabird foraging behavior.

## Teaser

Chick-rearing seabirds are more likely to mix short chick-feeding trips and long self-feeding trips when there are larger differences between habitat quality near and far from the breeding site. This strategy, called “dual foraging,” is common in seabirds feeding young, but its drivers are poorly understood. This study compiles results from over 100 studies to help clarify how environmental conditions and taxon-specific characteristics influence seabird feeding behavior.

## INTRODUCTION

Long-lived species must balance investment between current and future offspring to maximize their lifetime reproductive potential (Ricklefs 1977), based on predictions of life-history theory that investment in current offspring comes at a cost to parental survival and future reproductive success (Williams 1966; Charnov and Krebs 1974; Charlesworth 1980). This creates a conflict of interest between parents and offspring. For central-place foraging animals, this conflict is especially pronounced in individuals that feed at distant foraging patches (Abrams 1991; McNamara and Houston 1997) because the range and duration of foraging trips usually decrease with the onset of chick rearing, potentially putting the most productive foraging locations beyond reach (Weimerskirch et al. 1993). Understanding the roles of habitat quality and taxon-specific characteristics in chick-provisioning adults can provide valuable insights into how long-lived parents balance their own needs with the needs of their current offspring.

One tactic some long-lived animals use to solve this problem is dual foraging, which has mainly been found in birds, especially seabirds (Hernandez-Pliego et al. 2015; Hoskins et al. 2017; Fayet et al. 2021; Paiva et al. 2010a), but which has also been reported in other taxa, including seals (Skinner et al. 2012). First defined by Weimerskirch and colleagues (1993), the dual foraging strategy involves parents switching between short foraging trips to provision their offspring with long foraging trips to provision themselves. Short trips allow offspring to be fed regularly but are energetically costly for parents (Cuthill and Kacelnik 1990; Weimerskirch 1998; Weimerskirch and Cherel 1998), whereas long trips allow adults to forage primarily to maintain or recoup their own body condition while secondarily foraging for their offspring (Weimerskirch et al. 1994; Granadeiro et al. 1998). One reason long trips are unsuitable for offspring provisioning in birds is that, for most birds, the food would be digested by the time the parent returns to the nest. For birds that carry prey in their beaks, transporting the prey a long way may be impractical or costly and may also prevent the adult from feeding for prolonged periods. Both time and distance are important to the dual foraging concept; the energetic cost of travel is primarily determined by the distance to foraging sites, while chick survival is partly determined by the time between feeding events. Given these two perspectives, short and long trips have been defined in the dual foraging literature either in terms of distance from the colony (e.g., Weimerskirch et al. 1993) or duration away from the nest (e.g., Weimerskirch et al. 1994). As dual foraging is especially prevalent in seabirds, they are the focus of this review.

Theory predicts that chick-rearing seabirds will employ a mixture of short- and long-distance foraging trips when the resources near the colony are insufficient to provision both themselves and their offspring (Chaurand and Weimerskirch 1994; Granadeiro et al. 1998; Weimerskirch 1998; Weimerskirch and Cherel 1998). Supporting this theory, a few studies that report differences in the use of the dual foraging strategy in the same seabird population in different years (Irvine et al. 2000; Besel et al. 2018) posit that changes in the use of dual foraging may be due to changes in prey availability over time. Additionally, a review of the dual foraging literature in Procellariiformes by Baduini and Hyrenbach (2003) found that the long-distance foraging patches targeted by species that used dual foraging had higher concentrations of chlorophyll than short-distance foraging patches, which could be a marker indicating that the long-distance patches had greater prey availability. Furthermore, Shoji and colleagues (2015) found that the habitat quality of prey patches visited by chick-rearing Manx shearwaters *Puffinus puffinus* was highest at patches furthest from the colony.

Major advances in biologging technology since these foundational studies on dual foraging have enabled the emergence of a new generation of studies that track the foraging movements of chick-rearing seabirds with GPS loggers. While previous studies mainly estimated trip duration by measuring how long birds were away from their nest, these tracking studies provide data on the exact locations and distances foraging seabirds travel from their colonies. By combining GPS locations with remote-sensed environmental variables, it is possible to estimate the primary productivity of the habitats birds can access at different distances from the colony. Therefore, an updated review of the use of dual foraging strategies that integrates the results of these newer studies can shed light on the role of habitat quality in driving dual foraging.

Another critical issue which has not previously been systematically assessed is the potential role that differences in the quality of the habitat available during short and long foraging trips have in promoting (or discouraging) the use of a dual foraging strategy by different seabird taxa. Moreover, the potential moderating effect of species-specific differences, including differences in flight costs, whether chicks are fed with fresh or pre-digested prey, and the risk of chick starvation or predation between feeding visits, have not been systematically assessed. Cross-taxa analyses are needed to clarify similarities and differences in the determinants of the dual foraging strategy between different seabird families.

From a theoretical perspective, Ropert-Coudert and colleagues (2004) developed a model based on tracking data of chick-rearing Adélie penguin *Pygoscelis adeliae* which hypothesizes that adults adjust their foraging time and distance based both on their own stomach capacity and digestion rates and on that of their chicks. According to this model, there are occasions when alternatively using short and long foraging trips can maximize the net gain for adults and chicks. Olsson and colleagues (2014) developed a general habitat selection model based on the Central Place Foraging Theory which hypothesizes that a central place forager’s decision to choose a prey patch depends on whether foraging there would positively contribute to the animal’s fitness—which is a function of the quality of the patch and its distance from the colony. This model predicts that the maximum distance an animal is willing to travel to forage is a function of prey availability. So, when prey availability near the colony is poor, animals should be willing to travel further from the colony to forage. The model also predicts that the trade-offs different species make between patch quality and distance depend on their travel costs—species with low travel costs can profitably access patches at greater distances from the colony than species with high travel costs. However, the roles of travel costs and habitat quality near the colony on seabird foraging strategies have not been systematically assessed.

This analysis aims to address these outstanding questions with a review. To do so, we compiled all accessible studies in seabirds that provide information enabling us to identify the presence or absence of dual foraging—including both studies with tracking data and older studies without tracking data—and extracted data on the distance from the colony seabirds travelled on foraging trips, collecting results from 102 studies on 50 seabird species across 9 families. We then integrated these data with large-scale data on the chlorophyll-a concentration at different distances from their breeding colonies at the time the foraging data were collected. Using chlorophyll-a concentration as a proxy for habitat quality (Yen et al. 2006; Häkkinen et al. 2021; Frankish et al. 2022), we assessed the relationship between habitat quality around breeding colonies and the use of the dual foraging strategy during chick-rearing in different species of seabirds. More specifically, we tested whether the magnitude of the difference in habitat quality between locations close to and further from the colony (but still within reachable foraging distances) predicted the use of the dual foraging strategy and explored how this relationship differed between seabird families. Based on predictions from theoretical models (Houston and McNamara 1985; Cuthill and Kacelnik 1990; Waite and Ydenberg 1996), we hypothesized that the probability of dual foraging increases in parallel with increases in the magnitude of difference in the quality of foraging patches seabirds can access during short and long trips—in other words, that seabirds will be more likely to use dual foraging if there is more to be gained by foraging at greater distances.

## METHODS

### Literature search

We conducted a first literature search to identify all studies that consider dual foraging in seabirds. We used the Google Scholar search engine—which searches the full text of all available articles—using the search terms’ dual foraging’ (exact phrase) and “seabird” on 20 July 2020. After excluding patents and citations, this search yielded 348 unique results.

In addition, to identify the many studies which report unimodal or bimodal foraging trips of seabirds based on the duration or distance of foraging trips but do not specifically mention “dual foraging,” we conducted a second search on 14 August 2020, using the advanced search function in Google Scholar for articles that included the terms “seabird,” “foraging trip,” (exact phrase) and “bimodal” or “unimodal” while excluding papers that included the term “dual foraging” (exact phrase) (which were identified in the first search). This second search yielded an additional 388 unique results.

We used the search terms “bimodal,” “unimodal,” or “dual foraging” to refine our search, as our study required to know the distribution of foraging trip lengths to assess whether or not dual foraging occurred. Using wider search terms did not lead to a substantial change in the number of studies included (see Supplementary Materials).

### Inclusion and exclusion criteria

Of these 736 identified papers, those satisfying any of the following five criteria were included in the analysis.

1. The author(s) conclude that “dual foraging” does or does not occur during chick-rearing.
2. The author(s) conclude that the duration of foraging trips during chick-rearing was either “bimodal” or “unimodal.”
3. The author(s) conclude that the maximum distance from the colony of foraging trips during chick-rearing was “bimodal” or “unimodal.”
4. Short and long foraging trips during chick-rearing are analyzed separately based on previous studies which found that the same species conducts both short and long trips.
5. The author(s) present histograms of the duration or maximum trip distance of foraging trips during chick-rearing that visually demonstrate a clear unimodal or bimodal pattern (even if the authors do not discuss this).

We used the criteria above to include only data from studies that provided clear evidence about the presence or absence of dual foraging or about the unimodal or bimodal duration or maximum distance of foraging trips. In studies in which the data on the modality of the maximum distance of foraging trips and the maximum duration of foraging trips came to a different conclusion about the use of dual foraging, the final decision about the presence or absence of dual foraging was based on the results about the maximum distance of foraging trips. Papers were excluded if the presented data on duration and distance from the colony of foraging trips were not clearly stratified by breeding stage or if the authors’ assessment of the presence or absence of dual foraging and the raw data provided in the paper contradicted each other. We identified 102 papers which satisfied at least one of the criteria. These papers reported data collected between August 1987 and August 2018 from 50 seabird species across nine families in all six seabird orders (Supplementary Table S1).

### Data extracted

For each of those 102 selected papers (and for each separately reported colony in each paper), we extracted the following information: species studied, colony location (latitude and longitude), months and years of data collection (limited to the chick-rearing phase if data were collected in multiple breeding stages), data collection method(s) (e.g., GPS, isotope analysis, nest entrance monitoring, etc.), sample size (number of individuals studied), and whether or not dual foraging occurred (see Supplementary Table S1).

When a study included data from multiple colonies that were analyzed separately, we only included data from colonies for which the collected data met the inclusion and exclusion criteria. For multiple-year studies that included data from multiple chick-rearing seasons in which dual foraging occurred in some seasons but not in other seasons, we recorded data for the seasons in which dual foraging occurred separately from data for the seasons in which it did not occur. If multiple papers used the same datasets, we only included one paper, prioritizing peer-reviewed papers over theses and, secondarily, prioritizing papers that present more data from the dataset.

### Estimating distance from the colony of short and long foraging trips

For 22 of the 50 species of seabirds considered in the included papers, we recorded the mean distance of short and long foraging trips during chick-rearing reported in the paper or—if separate data were unavailable for short and long trips—the average distance of all foraging trips during chick-rearing. Papers about the remaining 28 species did not report either the distance of short and long trips or the overall mean of all foraging trips, so we searched for studies in Google Scholar (using the species name [exact phrase] and “distance to colony” as search terms) to identify studies that could provide this information. When information about trip distance during chick-rearing was unavailable, we considered studies that reported trip distance during breeding generally or during incubation. For 5 of the 50 species (Table 1), no information at all was available for the species about foraging trip distance during breeding, so we considered studies that reported trip distance during breeding in a closely related species based on the assumption that the maximum distance from the colony on foraging trips would be similar. When multiple distinct reports about foraging trip distances were identified for a particular species, we averaged the distances reported across colonies or studies (without weighting by sample size).

**Table 1:** Mean maximum distance to the colony of short- and long-distance trips for the 50 seabirds species considered

Species	Short trip distance (km)	Long trip distance (km)	Estimation method	Breeding stage^a^	Data from closely related species^b^	Source
Adélie penguin	35	98	min max	C	Not used	(Wienecke et al. 2000)
African penguin	8	33	min max	C	Not used	(Pichegru et al. 2010)
Antarctic prion	800	1546	SD	I	Thin-billed prion	(Quillfeldt et al. 2019)
Australasian gannet	20	50	In report	C	Not used	(Besel et al. 2018)
Barau’s petrel	150	1355	In report	C	Not used	(Pinet et al. 2012)
Black petrel	98	1128	min max	C	Not used	(Freeman et al. 2010)
Black-browed albatross	10	129	min max	C	Not used	(Arata et al. 2014)
Black-legged kittiwake	28	304	In report	C	Not used	(Christensen-Dalsgaard et al. 2018)
	29	71	In report	C	Not used	(Paredes et al. 2012)
	34	135	In report	C	Not used	(Paredes et al. 2012)
	37	64	In report	C	Not used	(Christensen-Dalsgaard et al. 2018)
	9	48	In report	C	Not used	(Kotzerka et al. 2010)
Blue petrel	800	1546	SD	I	Thin-billed prion	(Quillfeldt et al. 2019)
Blue-footed booby	8	46	SD	C+I	Not used	(Weimerskirch et al. 2009)
Brown pelicans	21	83	min max	I	Peruvian pelicans	(Zavalaga et al. 2011)
Cape gannet	29	322	min max	C	Not used	(Botha and Pistorius 2018)
Cape Verde shearwater	140	242	SD	C	Not used	(Paiva et al. 2015)
Chinstrap penguin	9	27	SD	C	Not used	(Kokubun et al. 2015)
Common diving petrel	21	33	SD	I	Not used	(Dunphy et al. 2020)
Common guillemots	34	134	SD	C	Not used	(Thaxter et al. 2012)
Cory’s shearwater	52	596	In report	C	Not used	(Paiva et al. 2010a)
	64	548	In report	C	Not used	(Paiva et al. 2010a)
	62	1060	In report	C	Not used	(Soares 2013)
Desertas petrel	1268	4690	min max	I	Murphy’s petrel	(Clay et al. 2019)
Flesh-footed shearwater	92	923	1/4, SD	C+I	Not used	(Waugh et al. 2016)
Gentoo penguin	3	11	In report	C	Not used	(Carpenter-Kling et al. 2017)
Great frigatebird	376	1022	min max	I	Not used	(Weimerskirch et al. 2010)
Great shearwater	299	2814	In report	C	Not used	(Schoombie et al. 2018)
Grey-headed albatross	399	2162	min max	C	Not used	(Xavier et al. 2003)
Humboldt penguin	7	30	min max	C	Not used	(Boersma et al. 2007)
Hutton’s shearwater	124	306	min max	C	Not used	(Bennet et al. 2019)
Laysan albatross	103	865	1/4, SD	C+I	Not used	(Montoya et al. 2019)
Light-mantled albatross	50	1650	min max	C	Not used	(Phillips et al. 2009)
Little auk	12	66	in report	C	Not used	(Jakubas et al. 2016)
	16	58	In report	C	Not used	(Jakubas et al. 2020)
Macaroni penguin	16	60	SD	C	Not used	(Trathan et al. 2006)
Magnificent frigatebird	26	928	1/4, max	C	Not used	(Austin et al. 2019)
Manx shearwater	19	1109	min max	C	Not used	(Wischnewski et al. 2019)
Nazca booby	98	329	In report	C	Not used	(Zavalaga et al. 2012)
Northern fulmar	154	646	SD	C	Not used	(Thaxter et al. 2012)
Northern gannet	79	268	SD	C	Not used	(Warwick-Evans et al. 2016)
Pink-footed shearwater	23	216	In report	C	Not used	(Carle et al. 2019)
Razorbill	3	73	1/4, max	C	Not used	(Isaksson et al. 2019)
Red-tailed tropicbird	27	317	SD	C	Red-billed tropicbirds	(Diop et al. 2018)
Scopoli’s shearwater	46	210	In report	C	Not used	(Pereira de Felipe 2020)
Short-tailed shearwater	130	312	min max	C	Not used	(Einoder and Goldsworthy 2005)
Shy albatross	24	267	min max	C	Not used	(Brothers et al. 1998)
Sooty shearwater	515	1970	In report	C	Not used	(Shaffer et al. 2009)
Streaked shearwater	15	775	min max	C	Not used	(Matsumoto et al. 2017)
Thick-billed murre	4	161	min max	C	Not used	(Jones et al. 2002)
Thin-billed prion	800	1546	SD	I	Not used	(Quillfeldt et al. 2019)
Wandering albatross	441	2192	In report	C	Not used	(Pereira et al. 2018)
Waved albatross	13	1397	min max	C+I	Not used	(Awkerman et al. 2014)
Wedge-tailed shearwater	69	262	In report	C	Not used	(Keys 2018)
Westland petrel	88	232	SD	C	Not used	(Waugh et al. 2018)
White-chinned petrel	62	1868	In report	C	Not used	(Catard et al. 2000)
Yellow-nosed albatross	11	594	min max	C	Not used	(Weimerskirch and Guionnet 2002)

C: chick-rearing stage; I: incubation; C+ I: during breeding (including incubation and chick-rearing);

min max: Short and long trip distances estimated from minimum and maximum trip distances

SD: Short-trip distance estimated as mean minus one standard deviation, long-trip distance estimated as mean plus one standard deviation

in report: Short and long trip distances are provided in the report of the study

1/4, SD: Long trip distance estimated as mean plus one standard deviation, short trip distance estimated at ¼ of mean trip distance

1/4, max: Long trip distance estimated as maximum trip distance, short trip distance estimated at ¼ of mean trip distance (see methods section for details)

^a^When species-specific distance to colony during the chick-rearing stage (C) was unavailable, either the distance from colony during breeding (incubation and chick-rearing, I+C) or the distance from colony during incubation (I) was used, depending on what data were available

^b^When unable to obtain species-specific distances to colony, distances to colony of closely related species were used

When the available reports only provided the overall mean maximum distance of foraging trips without stratifying results for short and long foraging trips, we estimated these values as follows. (1) If the study reported minimum and maximum trip distances, we used these as the mean maximum distances for short and long trips, respectively; this estimation method was used for 19 species (Table 1). (2) If the study only provided the overall mean trip distance with a corresponding standard deviation (or standard error) smaller than the mean, we estimated the mean maximum long-trip distance as the overall mean plus one standard deviation and the mean maximum short-trip distance as the overall mean minus one standard deviation (done for 13 species). (3) If the study only provided the overall mean trip distance with a corresponding standard deviation (or standard error) larger than the mean, we estimated the mean maximum long-trip distance as the overall mean plus one standard deviation and the mean maximum short-trip distance as ¼ of the overall mean trip distance (done for four species). We used this fraction of the mean to estimate short-trip distance because in the 19 studies of 12 species for which we had data on the mean distance of all trips and the distance travelled in both short and long foraging trips during chick-rearing, the mean maximum distance from the colony of short trips was approximately ¼ (0.241) of that of all foraging trips. (4) When a paper only presented the minimum and maximum or the mean and standard deviation of trip distances stratified by sex, colony, or season without an overall mean, we estimated the overall mean distances of short and long trips for the sample by averaging the corresponding values for the subsamples (without weighting for the size of the subsamples).

### Assessment of habitat quality

To estimate habitat quality available at different distances from the colonies in these studies, we used chlorophyll-a concentration as our metric of habitat quality. Nutrient-rich water promotes the primary production of phytoplankton (Abreu et al. 1995), which attracts zooplankton (Noges 1997), and in turn, zooplankton attracts the fish on which seabirds prey (Ware and Thomson 2005). Chlorophyll-a concentration is, therefore, a measure of primary production that provides an indirect estimate of the habitat quality of locations where seabirds forage. Numerous studies report that foraging seabirds depart colonies in specific directions (Trathan et al. 2006; Weimerskirch 2007), which suggests that they know where the best prey patches are likely to be located on a mesoscale. Thus, using the *maximum* chlorophyll-a concentration accessible within the short-distance foraging range and, separately, within the long-distance foraging range is probably a more accurate proxy for the quality of the prey patches available during short-distance and long-distance foraging than the *average* chlorophyll-a concentration within their short and long foraging ranges.

We extracted data on chlorophyll-a concentrations around colonies at the time the studies were conducted from the Global Ocean Biogeochemistry Hindcast dataset, downloaded from the Copernicus Marine Environment Monitoring Service (CMEMS 2020). The CMEMS dataset contained monthly means of global chlorophyll-a concentrations in mg.m^−3^ from 1 January 1993, to 23 December 2019, on a grid of 0.25 by 0.25 degrees. Dual foraging data during chick-rearing collected before these data were available in 1993 were excluded from this analysis; this resulted in excluding data on one of the 50 species considered (the common diving petrel *Pelecanoides urinatrix*) and excluding 80 of the 822 months of dual foraging data collected.

For each colony in each study, we calculated chlorophyll-a accessible on short trips as the maximum chlorophyll-a concentration within the radius of the species’ short-trip distance from the colony and chlorophyll-a accessible on long trips as the maximum chlorophyll-a concentration outside of the areas accessible during short trips but within the radius of long trips from the colony. The chlorophyll-a data were available as monthly averages, so we calculated the maximum chlorophyll-a concentrations available during short and long foraging trips from the colony for each month during which foraging data on chick-rearing seabirds were collected.

### Statistical analysis

To assess the relationship between seabirds’ use of the dual foraging strategy during chick-rearing and the habitat quality of short-distance and long-distance foraging sites, we used linear mixed effects models (LMMs) to test (1) whether locations with different chlorophyll-a concentrations were available during long and short trips in the months that chick-rearing seabirds employed dual foraging (model 1) and (2) whether the magnitude of the difference in chlorophyll-a concentrations in areas accessible during long versus short trips differed between the months in which dual foraging was and was not used (model 2). In model 1, the response variable was the maximum chlorophyll-a concentration accessible within the short-distance or long-distance foraging range. The fixed effect was the type of foraging trip (i.e., short or long). In model 2, the response variable was the *difference* in chlorophyll-a concentrations between areas accessible during short and long trips. The fixed effect was whether or not dual foraging was employed. For both models, we set species and study as random effects and square-root transformed chlorophyll-a concentration to normalize its distribution.

A third model employed a generalized linear mixed model (GLMM) to assess whether the probability of dual foraging changed with changes in the magnitude of the difference in habitat quality accessible during short and long trips. This model had a binomial family distribution: we set whether dual foraging occurred as the response variable, the difference in maximum chlorophyll-a concentrations accessible between short and long trips each month as a fixed effect, and seabird taxon as a random effect. We compared each of these three models with a null model (without the fixed effect) and used a likelihood ratio test to identify statistical significance.

We then built a generalized linear model (GLM) to assess differences in the use of dual foraging by different seabird taxa based on the difference in habitat quality available on short and long trips. This model had a binomial family distribution in which whether or not dual foraging occurred in the specified month was the binary response variable, and with the difference in maximum chlorophyll-a concentrations between the areas accessible during short and long trips each month and the family group of the species as fixed effects. We separated little auks *Alle alle* into their own group in the GLM analysis because their unique ability among auks to carry prey in a gular pouch (Stempniewicz 2001)—unlike other auks which carry fish in their beaks (e.g., Burke and Montevecchi 2009)—could result in the use of different foraging strategies.

## RESULTS

**Figure 1:**
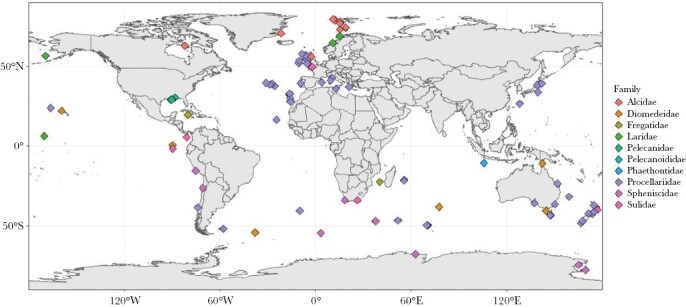
The location of study colonies reported in the 102 studies included in the analysis of dual foraging in seabirds.

We identified 102 studies which presented data that could be used to determine whether or not dual foraging occurred. These studies reported data on 50 species (Supplementary Table S1) across nine families: auks (Alcidae), albatrosses (Diomedeidae), frigatebirds (Fregatidae), gulls (Laridae), pelicans (Pelecanidae), tropicbirds (Phaethontidae), shearwaters (and prions and some petrels) (Procellariidae), penguins (Spheniscidae), and sulids (Sulidae), spanning all six orders containing seabirds (Charadriiformes, Procellariiformes, Suliformes, Pelecaniformes, Phaethontiformes, and Sphenisciformes). The colony locations reported in the 102 studies encompassed all oceans and continents (Figure 1). We estimated the mean maximum distances travelled from the colony on short-distance and long-distance foraging trips during chick-rearing for each of these 50 species (see Table 1). Trip distances spanned orders of magnitude across species, ranging from 3 km (Gentoo penguin *Pygoscelis papua,* short trips) to 4690 km (Desertas petrel *Pterodroma deserta,* long trips). Dual foraging was reported in 69 (67.6%) of the 102 studies, a combination of dual foraging and non-dual foraging (for different species, colonies, or years) was reported in 11 (10.8%) studies, and a lack of dual foraging was reported in 22 (21.6%) studies. In 2 of the 80 studies that reported dual foraging, dual foraging was only identified in males. Across the nine families to which the 50 species of seabirds considered here belong, dual foraging was found in all Phaethontidae and Laridae species, 80% of Sulidae species, 67% of Spheniscidae species, 55% of Procellariidae species, 50% of Alcidae and Fregatidae species, 25% of Diomedeidae species, and none of Pelecanidae species (see Table 2).

**Table 2:** Probability of dual foraging across nine seabird families

Family	Species that only used dual foraging (%)^a^	Species that sometimes used dual foraging (%)^a^	Species that never use dual foraging (%)^a^	Number of species	Number of studies
Laridae	100% (1)	0% (0)	0% (0)	1	2
Phaethontidae	100% (1)	0% (0)	0% (0)	1	1
Sulidae	80% (4)	0% (0)	20% (1)	5	5
Spheniscidae	67% (4)	16.6% (1)	16.6% (1)	6	10
Procellariidae	55% (12)	31% (7)	14% (3)	22	53
Alcidae	50% (2)	0% (0)	50% (2)	4	14
Fregatidae	50% (1)	0% (0)	50% (1)	2	2
Diomedeidae	25% (2)	37.5% (3)	37.5% (3)	8	10
Pelecanidae	0% (0)	0% (0)	100%	1	1

^a^Number in brackets indicates the number of species

While all regions may not be similarly well represented in our dataset, there was little difference in the use of dual foraging strategies between seabirds located in breeding colonies in different geographic regions: 68% of colonies in the tropics or subtropics (0–35°N or S, *n* = 41) used dual foraging, compared with 74% of colonies in temperate regions (35–50°N or S, *n* = 58), and 75% of colonies in subpolar or polar regions (>50°N or S, *n* = 52).

**Figure 2:**
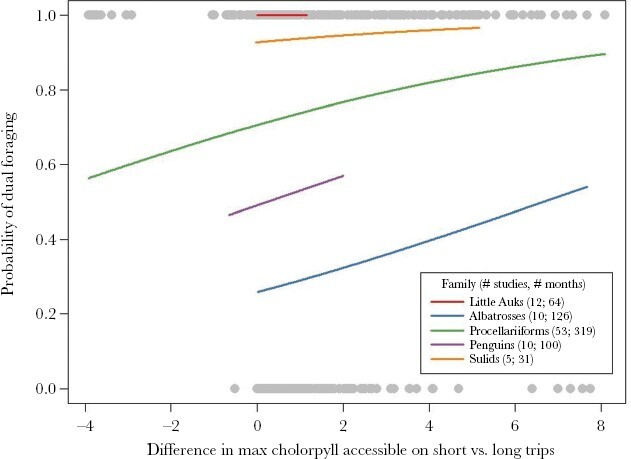
Probability of dual foraging during chick-rearing as a function of the difference in patch quality accessible during short and long foraging trips for five of the nine families of seabirds considered. Negative values on the X-axis indicate cases where the maximum chlorophyll-a concentrations within the area accessible for long trips (i.e., outside the area accessible during short trips) were poorer than the maximum chlorophyll-a concentrations in areas accessible during short trips. To avoid extrapolating beyond our dataset, the lines predicted from the generalized linear model are constrained (on the X-axis) to the chlorophyll-a differences recorded in that family. Grey dots are observed data points that indicate months when seabirds did (Y-axis = 1) and did not (Y-axis = 0) use the dual foraging strategy when the difference in patch quality available during short and long trips in the month was at the level specified by the X-axis coordinate of the dot.

Three results provided evidence that habitat quality is a driver of dual foraging in seabirds (Figure 2). First, in months when seabird species used dual foraging, chlorophyll-a concentrations accessible during long trips were significantly higher than those accessible during short trips (2.03 ± 0.08 vs. 0.95 ± 0.06, LMM: χ^2^_1_ = 298.28, *P* < 0.0001). Second, the difference in chlorophyll-a concentrations accessible between short and long trips was greater when dual foraging occurred than when it did not occur (1.08 ± 0.08 vs. 0.82 ± 0.08, LMM: χ^2^_1_ = 18.51, *P* < 0.0001). Third, the probability of dual foraging increased significantly with increases in the magnitude of the difference in habitat quality accessible between short and long trips (slope = 0.159 ± 0.059, GLMM: χ^2^_1_ = 7.55, *P* = 0.0060). In other words, seabirds were more likely to use a dual foraging strategy during chick-rearing when the habitat quality available further from the colony was much better than that available near the colony.

Using Procellariidae as the reference group, we found that after accounting for differences in habitat quality available during short and long foraging trips, Alcidae (excluding *Alle alle*), Diomedeidae, and Spheniscidae, were significantly less likely to use the dual foraging strategy than Procellariidae, while Sulidae were significantly more likely to use the dual foraging strategy than Procellariidae. There were no statistically significant differences in the probability of using the dual foraging strategy between the remaining families (*Alle alle*, Fregatidae, Laridae, Pelecanidae, Phaethontidae) and Procellariidae (see Table 3).

**Table 3:** Results of generalized linear model with a binomial family distribution^a^

Seabird family	Estimate	Standard error	z value	*P*-value
Intercept	0.8766	0.1424	6.1551	**7.50E-10**
Procellariidae (reference family)	0.1585	0.0594	2.6663	**0.0077**
Alcidae	−1.4055	0.3162	−4.4457	**8.76E-06**
Alle alle	17.6325	815.0714	0.0216	0.9827
Diomedeidae	−1.9319	0.2351	−8.2157	**2.11E-16**
Fregatidae	−0.2351	0.8766	−0.2682	0.7885
Laridae	17.5909	1190.4050	0.0148	0.9882
Pelecanidae	−19.5959	2300.8680	−0.0085	0.9932
Phaethontidae	17.3559	3255.7320	0.0053	0.9957
Spheniscidae	−0.9137	0.2426	−3.7657	**0.0002**
Sulidae	1.6771	0.7433	2.2562	**0.0241**

^a^The model uses the difference in maximum chlorophyll concentrations between the area accessible during short and long trips in each month as a fixed effect, the family group of the seabird species as a fixed effect, and whether or not dual foraging occurred in that month as the binary response variable

## DISCUSSION

By integrating the results of 102 studies of seabird foraging during chick-rearing with estimates of habitat quality near the colony, our study shows that (1) dual foraging during chick-rearing is common among seabirds, and (2) the probability of dual foraging across seabird families increases with increasing differences in foraging habitat quality between close and far prey patches, but with differences between families.

### Differences in dual foraging across seabird families

Dual foraging occurred in 50–100% of seabird species in eight of the nine families included in the review; the exception was the Pelecanidae family, but we only located a single study for this family, so their propensity to use dual foraging should be re-assessed in subsequent studies. The propensity for dual foraging varied between families: auks (excluding little auks), albatrosses, and penguins were all significantly less likely to use the dual foraging strategy during chick-rearing than Procellariidae, while Sulids were significantly more likely to use the dual foraging strategy than Procellariidae.

In some seabirds, chicks accumulate such large amounts of fat that their mass exceeds that of their parents. This is particularly evident in Procellariiformes (albatrosses, shearwaters, and petrels), among which some chicks attain a mass 60% greater than that of their parents (Warham 1990), though they subsequently lose this fat before fledging (Lack 1968; Ricklefs et al. 1980; Warham 1990). Building on an earlier theory by Lack (1968), Ricklefs and Schew (1994) hypothesized that these fat deposits were a result of parental over-feeding, a strategy that provides chicks with enough resources to weather the natural stochastic variation in food deliveries. This hypothesis is supported by empirical evidence from a study of little shearwaters *Puffinus assimilis* which showed that the accumulation of fat in chicks was related to the irregular timing of parental food delivery (Hamer 1994). These fat deposits likely buffer shearwater, albatross, and petrel chicks during extended periods without feeding. The additional fat stores in these chicks may also promote high-quality feather growth over a relatively short period, which is energetically demanding. This excess fat deposit hypothesis may help explain why dual foraging is common in Procellariidae. However, it does not explain why albatrosses were the least likely to use the dual foraging strategy amongst the five seabird families in which we had enough power to detect a significant difference (Alcidae, Diomedeidae, Procellariidae, Spheniscidae, and Sulidae). One possible explanation for this unexpected result is that albatrosses have longer chick-rearing periods than most other seabirds. Hence, the period where albatross chicks are very young and most dependent on frequent feedings is a smaller part of the total chick-rearing period than other seabirds. Thus dual foraging may be a less important strategy for albatrosses after their chicks reach a certain age, after which the relative benefit of short trips near the colony would be smaller. If this were true, we would expect albatrosses to dual forage more at the beginning of chick rearing and less later on in chick rearing.

Auks (excluding little auks) were also not very likely to employ dual foraging. There are several possible explanations. First, auks have a high flight cost due to their high wing loading (Pennycuick 1987), so they must expend much more energy than other birds as travel distances increase (Gaston 1985). This limitation would make them likely to adopt foraging strategies that minimize flight time, only foraging in prey patches further from the colony when those immediately around the colony become severely depleted—as often occurs around large colonies (Elliott et al. 2009). Moreover, unlike Procellariidae, which regurgitate food for their chicks (Prince and Morgan 2009), most auks carry prey to chicks in their beak, restricting the amount of food they can bring back from each trip; this may require parents to stay close to the colony to ensure frequent food deliveries. Finally, auk chicks have lower nestling obesity than Procellariidae (Visser 2001), making them more susceptible to starvation during extended periods without feeding. These factors may explain our finding that auks (other than little auks) are significantly less likely to use the dual foraging strategy than Procellariidae.

The distances from the colony penguins travelled on short and long foraging trips tended to be on the lower end of the foraging distances of all seabirds included in this analysis (Table 1), presumably due to the smaller distances reachable through swimming compared to flying. These shorter foraging trips of penguins may also be related to the availability of sufficient food resources at shorter distances from penguin colonies (reducing the need to travel further). In support of the latter theory, temperate and polar waters are more productive and more predictable than tropical waters (Longhurst and Pauly 1987; Weimerskirch 2007), so the polar waters in which penguin colonies are located are more likely to have sufficient resources to provision both parents and offspring near the colony than the waters surrounding colonies of seabirds breeding in the tropics. This difference may partially explain why penguins are less likely to use the dual foraging strategy than Procellariidae.

### Influence of habitat quality on the probability of dual foraging

Baduini and Hyrenbach’s review (2003) of dual foraging in Procellariiformes found that chlorophyll-a concentrations were higher in areas Procellariiformes targeted on long trips than in areas they targeted during short trips. Our results show that this is also the case in other families of seabirds; when seabirds used the dual foraging strategy, the chlorophyll-a concentrations at foraging sites accessible during long trips were significantly higher than that at sites accessible during short trips. Moreover, we found that as the magnitude of the difference in the chlorophyll-a concentrations between areas accessible during long and short trips increases, the likelihood of using the dual foraging strategy also increases. Although there were differences between families, the fact that we found this trend across numerous seabird families provides evidence that habitat quality is an important driver of dual foraging.

One difference between Baduini and Hyrenbach’s review (2003) and our study is that we found that albatrosses were significantly less likely to dual forage than Procellariidae, whereas they found no differences in the use of dual foraging across tube-nosed families. While Baduni and Hyrenbach classified foraging reported in studies in which they could not identify “distinct bimodality” as “unimodal,” we only included papers in which there was clear evidence of unimodal or bimodal foraging. This difference in inclusion criteria may partly explain our differing results.

While the discrepancy in the locations of the most productive foraging grounds and the colony may be a pre-requisite for the use of the dual foraging strategy (this study; Chaurand and Weimerskirch 1994; Weimerskirch et al. 1994; Granadeiro et al. 1998; Welcker et al. 2009; Tyson et al. 2017), the timing and frequency of short and long trips are likely driven by the condition of the parent and the chick (Weimerskirch 1998; Weimerskirch and Cherel 1998; Ochi et al. 2010; Wischnewski et al. 2019). In some cases, the type of trip one parent undertakes may also be influenced by the preceding trip of the other parent (Tyson et al. 2017). However, whether the needs of the parents, offspring, or both, determine foraging trip distance is far from clear (Wischnewski et al. 2019). Studies have found that whether adults feed close to or far from the colony is either determined solely by adult condition (short-tailed shearwaters *Puffinus tenuirostris* [Weimerskirch and Cherel 1998]; sooty shearwater *Ardenna grisea* [Weimerskirch 1998]), solely by chick condition (Manx shearwaters [Wischnewski et al. 2019]) or by both adult and chick condition (Streaked shearwater *Calonectris leucomelas* [Ochi et al. 2010]). This is an important question that needs further research across seabird taxa.

### Limitations

As our literature search was restricted to studies that mentioned “dual foraging,” “unimodal” or “bimodal,” our dataset might be biased toward certain seabird families if studies are looking for dual foraging in species which are most expected to employ this strategy. Also, as all seabird families have not been equally tracked, there may be imbalances in our dataset. However, our dataset still represents most seabird families.

While it would have been interesting to investigate how productivity around different breeding colonies might affect foraging distances within a species, we only had sufficient data for seven of the 50 species and found no significant effects (see Supplementary Materials).

Other factors not considered in this study may influence the likelihood of seabirds dual foraging. First, we did not consider whether the target species feed their offspring with prey different from what they consume, a factor that could increase the likelihood of dual foraging. This information is unknown for too many species to have been included in this study. Second, few studies reported the distance from the colony that seabirds travelled for both short and long foraging trips. Therefore, we had to estimate short-trip and long-trip foraging distances for many species. This may have decreased the precision of the distance estimates used in our analysis. Another limitation is using maximum chlorophyll-a concentration as a proxy for prey availability. This method has been used in several other studies (e.g., Ainley et al. 2009; Campbell et al. 2009; Marrari et al. 2019), but it may be misleading in some situations (Gremillet et al. 2008). For example, in cases where seabirds in large colonies deplete prey near the colony, the “real” differences in patch quality available during short- and long-distance foraging may be larger than that reflected by the chlorophyll-a concentration. We made this approximation because we do not have direct data on prey availability for most of these locations.

## CONCLUSION AND FUTURE DIRECTIONS

Overall, this study advances our understanding of the environmental drivers of dual foraging and how they interact with seabird taxa. We show that the probability of dual foraging across seabird families increased with increasing differences in maximum chlorophyll-a concentrations accessible during short versus long foraging trips, suggesting a within-species flexibility that could be the result of responding to these differences in real-time, which would be a mechanism or behavioral trigger instead of an evolutionary response. We also show differences between seabird families in the likelihood of dual foraging after controlling for habitat quality, suggesting a functional driver of dual foraging. Chick obesity, flight costs, and the method parents use to deliver food to chicks may influence the likelihood of dual foraging. This analysis provides evidence to help us understand a core question about seabirds’ foraging decisions: how do environmental conditions and taxa jointly influence foraging decisions?

Future studies should integrate large-scale data on fronts and other oceanographic features with chlorophyll-a concentration data to develop a more nuanced, time- and location-specific metric of prey abundance to understand drivers of dual foraging in greater detail. They should also take into account whether estimates of prey abundance near colonies based on chlorophyll-a concentrations need to be adjusted based on information about colony size and the likelihood that over-feeding has depleted prey abundance near the colony. Finally, the family-specific and species-specific factors that influence the use of the dual foraging strategy during chick-rearing (including flight costs, chick fat content, risk of predation, etc.) of different seabird families and species should be investigated further; this work will help clarify the relative roles of external environmental stimuli (primarily prey availability) and internal genetic and biological determinants in the selection and molding of seabirds’ foraging strategies.

## Supplementary Material

arad052_suppl_Supplementary_Materials

arad052_suppl_Supplementary_Table_S1

## Data Availability

Analyses reported in this article can be reproduced using the data provided by Phillips et al. (2023).
