# Supplementary material for: How do resource distribution and taxonomy affect the use of dual foraging in seabirds? A review
Source: Behav Ecol. 2023 Jul 11;34(5):769–79. doi: 10.1093/beheco/arad052 (PMC10516677; doi:10.1093/beheco/arad052)
Supplement: arad052_suppl_Supplementary_Materials [file arad052_suppl_supplementary_materials.docx]

**Supplementary Materials for:**

Phillips J.A., Guilford T. & Fayet A.L. (2023) How do resource distribution and taxonomy affect the use of dual foraging in seabirds?: a review. *Behavioral Ecology*

**1 - Impact of using wider search terms for the literature search**

To assess how many studies we might have missed by using our search terms compared to wider search terms without any mention of dual foraging, unimodality or bimodality, we conducted a new literature search on Google Scholar, using the search terms "seabird", "foraging trip", and "chick feeding" on February 10^th^, 2023, and evaluated the first 25 results. Of those 25 results, 12 did not provide data we could use, eight were already included in our review, and one was a book chapter we could not access (see Table S1). Three had data we could use but were not included in our study. Of these three, one was published after the search for our review was performed and did mention dual foraging, so our previous search would have picked it up had the dates aligned. Therefore, only two out of 25 results (8%) could have brought in more data into our review by widening the search terms. We are therefore confident our initial search terms give a representative overview of the relevant literature.

**2 – Interpopulation differences in foraging distance**

We attempted to conduct an analysis to investigate how habitat productivity around different populations would affect foraging distances/modalities within the same species. Unfortunately, of the 50 species for which we had data, data from multiple colonies were available for nine species only.

We considered using a binomial model that predicts the likelihood of dual foraging based on species and local productivity, but there were few instances of no dual foraging in this dataset. So instead, we focused only on the species and colonies with dual foraging and looked at whether the difference between short and long trip distances could be predicted by the difference in chlorophyll-a between far and close locations around each colony.

After removing the sites where there was no dual foraging, we were left with seven species that had data for multiple colonies. We ran a linear mixed model, with the difference in distance between short and long trips as a response variable and the difference in CHL between short and long trips as a predictor variable, with colony and year as random effects. We found no significant effect (slope=0.382±2.16, LMM: χ^2^_1_ = 0.0317, p=0.859).

**Table S1.** Assessment of first 25 results of literature search using wider search terms

| **First author** | **Year** | **Title** | **Assessment** |
| --- | --- | --- | --- |
| Chaurand | 1994 | The Regular Alternation of Short and Long Foraging Trips in the Blue Petrel Halobaena caerulea: A Previously Undescribed Strategy of Food Provisioning in a Pelagic Seabird | already included in our review |
| Paredes | 2012 | Proximity to multiple foraging habitats enhances seabirds' resilience to local food shortages | already included in our review |
| Weimerskirch | 1998 | How can a pelagic seabird provision its chick when relying on a distant food resource? Cyclic attendance at the colony, foraging decision and body condition in sooty… | already included in our review |
| Weimerskirch | 2003 | Foraging efficiency and adjustment of energy expenditure in a pelagic seabird provisioning its chick | already included in our review |
| Weimerskirch | 1999 | Natural and experimental changes in chick provisioning in a long-lived seabird, the Antarctic prion | already included in our review |
| Weimerskirch | 1995 | Adjustment of parental effort to manipulated foraging ability in a pelagic seabird, the thin-billed prion Pachyptila belcheri | already included in our review |
| Welcker | 2009 | Sex‐specific provisioning behaviour in a monomorphic seabird with a bimodal foraging strategy | already included in our review |
| Wojczulanis-Jakubas | 2018 | Seabird parents provision their chick in a coordinated manner | already included in our review |
| Shealer | 2002 | Foraging behavior and food of seabirds | inaccessible |
| Barger | 2016 | Resource partitioning between sympatric seabird species increases during chick‐rearing | missed by our initial search |
| Quillfeldt | 2010 | Inter-annual changes in diet and foraging trip lengths in a small pelagic seabird, the thin-billed prion Pachyptila belcheri | missed by our initial search |
| Burke | 2009 | The foraging decisions of a central place foraging seabird in response to fluctuations in local prey conditions | no usable data |
| Camphuysen | 2015 | Sexually distinct foraging strategies in an omnivorous seabird | no usable data |
| Cianchetti-Benedetti | 2015 | A new algorithm for the identification of dives reveals the foraging ecology of a shallow-diving seabird using accelerometer data | no usable data |
| Gauthier-Clerc | 2002 | Seabird reproduction in an unpredictable environment: how king penguins provide their young chicks with food | no usable data |
| Gremillet | 2015 | Arctic warming: nonlinear impacts of sea‐ice and glacier melt on seabird foraging | no usable data |
| Grissot | 2019 | Parental coordination of chick provisioning in a planktivorous arctic seabird under divergent conditions on foraging grounds | no usable data |
| Lewis | 2001 | Evidence of intra-specific competition for food in a pelagic seabird | no usable data |
| Navarro | 2007 | Experimental increase of flying costs in a pelagic seabird: effects on foraging strategies, nutritional state and chick condition | no usable data |
| Piatt | 2007 | Seabirds as indicators of marine food supplies: Cairns revisited | no usable data |

**Table S1.** Assessment of first 25 results of literature search using wider search terms (continued)

| **First author** | **Year** | **Title** | **Assessment** |
| --- | --- | --- | --- |
| Wojczulanis-Jakubas | 2022 | Post-foraging in-colony behaviour of a central-place foraging seabird | no usable data |
| Irons | 1998 | Foraging area fidelity of individual seabirds in relation to tidal cycles and flock feeding | no usable data |
| Kidawa | 2015 | Parental efforts of an Arctic seabird, the little auk Alle alle, under variable foraging conditions | no usable data |
| Thaxter | 2009 | Sex‐specific food provisioning in a monomorphic seabird, the common guillemot Uria aalge: nest defence, foraging efficiency or parental effort? | no usable data |
| Fayet | 2021 | Local prey shortages drive foraging costs and breeding success in a declining seabird, the Atlantic puffin | published after our initial search but would have been included |
