# Supplementary material for: How do resource distribution and taxonomy affect the use of dual foraging in seabirds? A review
Source: Behav Ecol. 2023 Jul 11;34(5):769–79. doi: 10.1093/beheco/arad052 (PMC10516677; doi:10.1093/beheco/arad052)
Supplement: arad052_suppl_Supplementary_Table_S1 [file arad052_suppl_supplementary_table_s1.docx]

| **Table S1.** Data extracted from studies included in the review | | | | | |  |
| --- | --- | --- | --- | --- | --- | --- |
| **Species** | **Colony location** | **Data collection years*** | **Study method** | **Dual foraging?** | **Citation** | |
| Adélie penguin | Bechervaise Island, East Antarctica | 1991-1992, 1995-1996 | weigh bridge | Y | (Clarke et al. 1998) | |
|  |  | 1991-2003 | PTT | Y | (Clarke et al. 2006) | |
|  |  | 1993-1994 | nest attendance | N | (Irvine et al. 2000) | |
|  |  | 1998-1999 | nest attendance | Y | (Irvine et al. 2000) | |
|  | Edmonson Point, Ross Sea | 1994-1996 | weigh bridge | N | (Clarke et al. 1998) | |
|  | Ross Island, Antarctica | 1997-2007 | weigh bridge | N | (Ballard et al. 2010) | |
| African penguin | Robben Island, South Africa | 2011-2013 | GPS | N | (Campbell 2016) | |
| Antarctic prion | Île Verte, Kerguelen islands, Southern Indian Ocean | 1995-1996 | nest attendance, chick weighing | Y | (Weimerskirch et al. 1999) | |
| Australasian gannet | Hawke's Bay, New Zealand | 2007-2009 | GPS, blood | Y | (Besel et al. 2018) | |
| Barau's petrel | Reunion Island, Indian Ocean | 2008-2009 | GLS | Y | (Pinet et al. 2012) | |
| Black petrel | Mount Hobson, New Zealand | 2006 | GPS | Y | (Freeman et al. 2010) | |
| Black-browed albatross | Bird Island, South Georgia | 1990,1992-1994, 2002 | radio transmitter, PTT | N | (Phillips et al. 2009) | |
|  | Kerguelen island, Southern Indian Ocean | 1992 | nest attendance, adult weighing | N | (Weimerskirch et al. 1994) | |
| Black-legged kittiwake | Sor-Gjaeslingan, central Norwegian sea | 2011-2014 | GPS | Y | (Christensen-Dalsgaard et al. 2018) | |
|  | Anda, northern Norwegian Sea | 2011-2014 | GPS | Y | (Christensen-Dalsgaard et al. 2018) | |
|  | St. George, south-eastern Bering sea | 2009 | GPS, activity loggers, blood, diet | Y | (Paredes et al. 2012) | |
|  | The Pribilof Islands, Bering sea | 2009 | GPS, activity loggers, blood, diet | Y | (Paredes et al. 2012) | |
| Blue petrel | Mayes Island, Kerguelen Islands, Southern Indian Ocean | 1990 | nest attendance | Y | (Chaurand and Weimerskirch 1994) | |
|  |  | 1990-2000 | Chick and adult weighing | Y | (Weimerskirch et al. 2003) | |
|  |  | 1998 | nest attendance | Y | (Cherel et al. 2002) | |
|  | Kerguelen islands, Southern Indian Ocean | 1989 | nest attendance, adult weighing | Y | (Weimerskirch et al. 1994) | |

| **Table S1.** Data extracted from studies included in the review (continued) | | | | | |
| --- | --- | --- | --- | --- | --- |
| **Species** | **Colony location** | **Data collection years*** | **Study method** | **Dual foraging?** | **Citation** |
| Blue-footed boobie | Isla Lobos de Tierra, northern Peru | 2002-2003 | Depth logger, flight sensor, diet | Y | (Zavalaga et al. 2007) |
| Brown Pelican | Gaillard Island, Alabama, USA | 2012 | GPS | N | (Walter et al. 2014) |
|  | Raccoon island, Louisiana, USA | 2012 | GPS | N | (Walter et al. 2014) |
|  | Shallow Bayou island, Louisiana, USA | 2012 | GPS | N | (Walter et al. 2014) |
|  | Queen Bess Island, Louisiana, USA | 2012 | GPS | N | (Walter et al. 2014) |
| Cape gannet | Bird Island, Algoa Bay, South Africa | 1994-1995 | activity meter, stomach contents | Y | (Adams and Klages 1999) |
| Cape verde shearwater | Raso Islet, Cape Verde | 2013 - 2014 | GPS | Y | (Paiva et al. 2015) |
| Chinstrap penguin | Nyroysa, west coast of Bouvetoya | 2007-2008 | PTT, TDR | Y | (Blanchet et al. 2013) |
| Common guillemot | Isle of May, south-east Scotland | 1999, 2001-2003, 2005 | activity logger or TDR | N | (Thaxter et al. 2010) |
| Cory's Shearwater | Corvo Island, Azores archipelago, North Atlantic | 2004-2006 | radio transmitter, PPT, blood | Y | (Magalhaes et al. 2008) |
|  |  | 2007 | GPS | Y | (Paiva et al. 2010a) |
|  |  | 2007 | compass-temperature, TDR | Y | (Paiva et al. 2010b) |
|  |  | 2010 | GPS | N | (Ceia et al. 2015) |
|  |  | 2010 | GPS, isotope | N | (Ceia 2013) |
|  | Cima Islet, Porto Santo Island, North Atlantic | 2011 - 2012 | GPS, isotope | Y | (Soares 2013) |
|  |  | 2011-2012, 2014-2015 | GPS | Y | (Avalos et al. 2017) |
|  | Selvagem Grande, Selvagens, North Atlantic | 1997 | nest attendance, chick weighing | Y | (Granadeiro et al. 1998) |
|  |  | 2007-2008 | compass-temperature, TDR | Y | (Paiva et al. 2010b) |
|  |  | 2008 | GPS | Y | (Paiva et al. 2010a) |
|  |  | 2010 | GPS, diet | N | (Alonso et al. 2012) |

| **Table S1.** Data extracted from studies included in the review (continued) | | | | | |  |
| --- | --- | --- | --- | --- | --- | --- |
| **Species** | **Colony location** | **Data collection years*** | **Study method** | **Dual foraging?** | **Citation** | |
| Cory's Shearwater | Berlengas islands, Portugal | 1987 | nest attendance, chick weighing | N | (Granadeiro et al. 1998) | |
|  |  | 2005-2007 | compass-temperature, TDR | Y | (Paiva et al. 2010b) | |
|  |  | 2007 | GPS | N | (Paiva et al. 2010a) | |
|  |  | 2011-2012, 2014-2015 | GPS | N | (Avalos et al. 2017) | |
|  | Faial, Azores archipelago, North Atlantic | 2006 | compass-temperature, TDR | Y | (Paiva et al. 2010b) | |
|  | Vila Islet, Azores archipelago, North Atlantic | 2004-2006 | radio transmitter, PPT, blood | Y | (Magalhaes et al. 2008) | |
|  | Praia Islet, Azores archipelago, North Atlantic | 2004-2006 | radio transmitter, PPT, blood | Y | (Magalhaes et al. 2008) | |
|  | Gran Canaria Island, Canary Island | 2005 | PTT, isotope | N | (Navarro and Gonzalez-Solis 2009) | |
|  | Desertas, Madeira archipelago | 2006 | compass-temperature, TDR | Y | (Paiva et al. 2010b) | |
| Desertas petrel | Bugio island, Madeira archipelago | 2007-2009 | GLS | Y | (Ramirez et al. 2013) | |
| Common diving petrel | Kerguelen island, Southern Indian Ocean | 1992 | Nest attendance, adult weighing | N | (Weimerskirch et al. 1994) | |
| Flesh-footed shearwater | Lord Howe Island, Australia | 2004-2005 | nest attendance, light and temperature loggers, chick weighing | Y | (Thalmann et al. 2010) | |
| Fulmar | Hirta, St. Kilda, Scotland | 1994 | chick weighing | N | (Hamer and Thompson 1997) | |
| Gentoo penguin | Trypot Beach, Marion Island, subantarctic Indian Ocean | 2014 | GPS, TDR | Y | (Carpenter-Kling et al. 2017) | |
| Great frigatebird | Europa Island, Mozambipue Channel | 2003 | PTT, altimeters, diet | N | (Weimerskirch et al. 2004) | |
| Great shearwater | Gough Island, Atlantic Ocean | 2014 | GPS, TDR | Y | (Schoombie et al. 2018) | |
| Grey-headed albatross | Marion Island, subantarctic Indian Ocean | 1998 | PTT, GLS | Y | (Nel et al. 2000)^ʎ^ | |
|  | Bird Island, South Georgia | 1993-1996, 2001 | radio transmitter/ PTT | N | (Phillips et al. 2009) | |
| Humboldt penguin | Punta San Juan, Peru | 1999 | TDR | Y | (Taylor et al. 2002) | |
|  | Isla Pan de Azucar, northern Chile | 1994 - 1995 | TDR | Y | (Luna-Jorquera 1999) | |

| **Table S1.** Data extracted from studies included in the review (continued) | | | | | |  |
| --- | --- | --- | --- | --- | --- | --- |
| **Species** | **Colony location** | **Data collection years*** | **Study method** | **Dual foraging?** | **Citation** | |
| Hutton's shearwater | Kaikoura Peninsula, New Zealand | 2017-2018 | GPS, TDR | N | (Bennet et al. 2019) | |
| Laysan albatross | Kilauea Point, Hawaii | 2007 | radio transmitters | Y | (Sprague 2009) | |
| Light-mantled albatross | Bird Island, South Georgia | 2003 | radio transmitter/ PTT | N | (Phillips et al. 2009) | |
| Little auk | Hornsund, southwest Spitsbergen, Norway | 2004, 2006 | nest attendance | Y | (Wojczulanis-Jakubas et al. 2010) | |
|  |  | 2004, 2016, 2017 | nest attendance | Y | (Wojczulanis-Jakubas et al. 2020) | |
|  |  | 2007 | TDR, nest attendance | Y | (Welcker et al. 2009a)^Ʈ^ | |
|  |  | 2009 - 2010 | GPS, temperature loggers | Y | (Kidawa et al. 2012) | |
|  |  | 2011, 2016, 2018 | GPS | Y | (Jakubas et al. 2020) | |
|  | Magdalenefjorden, northwest Spitsbergen, Norway | 2008-2010 | nest attendance, chick diet | Y | (Hovinen et al. 2014) | |
|  |  | 2009 | temperature loggers | Y | (Jakubas et al. 2014) | |
|  |  | 2009 - 2010 | GPS, temperature loggers | Y | (Kidawa et al. 2012)^ǂ^ | |
|  |  | 2009, 2010 | nest attendance | Y | (Wojczulanis-Jakubas et al. 2020) | |
|  |  | 2009-2010 | nest attendance | Y | (Wojczulanis-Jakubas et al. 2018) | |
|  | Kongsfjorden, Norway | 2007 | TDR, nest attendance | Y | (Welcker et al. 2009a)^Ʈ^ | |
|  |  | 2006- 2007 | nest attendance | Y | (Welcker et al. 2009b) | |
|  | Bjørndalen, Isfjorden, Norway | 2005 | nest attendance, plankton survey | Y | (Steen et al. 2007) | |
|  |  | 2006- 2007 | nest attendance | Y | (Welcker et al. 2009b) | |
|  |  | 2007 | TDR, nest attendance | Y | (Welcker et al. 2009a) | |
|  |  | 2008 | nest attendance, weighing | Y | (Welcker et al. 2012) | |
|  |  | 2008- 2010 | nest attendance, chick diet | Y | (Hovinen et al. 2014) | |
|  | Kap Høegh, Greenland | 2007 | TDR, nest attendance | Y | (Welcker et al. 2009a) | |
|  | Bear Island (Bjørnøya), Norway | 2007 | TDR, nest attendance | Y | (Welcker et al. 2009a) | |
|  |  | 2013 | GPS | Y | (Jakubas et al. 2016) | |
| Macaroni penguin | Nyroysa, west coast of Bouvetoya | 2007-2008 | PTT, TDR | Y | (Blanchet et al. 2013) | |
|  | Bird Island, South Georgia | 1997-2000 | radio transmitters, diet | Y | (Barlow and Croxall 2002) | |

| **Table S1.** Data extracted from studies included in the review (continued) | | | | | |  |
| --- | --- | --- | --- | --- | --- | --- |
| **Species** | **Colony location** | **Data collection years*** | **Study method** | **Dual foraging?** | **Citation** | |
| Magnificent frigatebird | Little Cayman, Cayman islands | 2017 | GPS, GPS-GSM, bird-borne camera, diet | Y | (Austin et al. 2019) | |
| Manx shearwater | Skomer Island, southwest Wales | 1999 | nest attendance | N | (Gray and Hamer 2001) | |
|  |  | 2002 | meal size, feeding frequency | N | (Hamer et al. 2006) | |
|  |  | 2003 | GPS, TDR | Y | (Shoji et al. 2015) | |
|  |  | 2009-2011 | GPS | Y | (Dean et al. 2015) | |
|  | Copeland, Northern Ireland | 2009-2011 | GPS | Y | (Dean et al. 2015) | |
|  | Lundy, England | 2009-2011 | GPS | Y | (Dean et al. 2015) | |
|  | Rum, Scotland | 2009-2011 | GPS | Y | (Dean et al. 2015) | |
|  | Great Balsket, Ireland | 2014-2015 | GPS, nest attendance | Y | (Wischnewski et al. 2019) | |
|  | High Island, Ireland | 2014 | GPS, nest attendance | Y | (Wischnewski et al. 2019) | |
| Nazac boobie | Punta Cevallos, Isla Espanola | 2007 | GPS, TDR | N | (Zavalaga et al. 2012) | |
| Northern gannet | Les Etacs, Alderney | 2011,2013-2015 | GPS | Y | (Warwick-Evans et al. 2016) | |
| Pink-footed Shearwater | Isla mocha, Chile | 2015-2017 | GPS | Y | (Carle et al. 2019) | |
| Razorbill | Isle of May, southeast Scotland | 1999, 2002, 2003, 2005, 2006 | activity logger, TDR | Y | (Thaxter et al. 2010) | |
| Red-tailed tropicbird | Christmas island, Indian Ocean | 2006 | nest attendance, immersion loggers, TDR | Y | (Sommerfeld and Hennicke 2010) | |
| Scopoli's shearwater | island of Linosa, Mediterranean | 2008, 2009, 2012 | GPS | Y | (Cecere et al. 2014) | |
|  | Tuscan Archipelago, Mediterranean | 2010, 2011 | GPS | N | (Cecere et al. 2014) | |
|  | La Maddalena Archipelago, Mediterranean | 2013 | GPS | N | (Cecere et al. 2014) | |
|  | Strofades Island complex, Ionian sea | 2014 | GPS | N | (Karris et al. 2018) | |
|  | Menorca Island, Cala Morell, Menorca | 2012-2015 | GPS | Y | (Pereira de Felipe 2020) | |

| **Table S1.** Data extracted from studies included in the review (continued) | | | | | |  |
| --- | --- | --- | --- | --- | --- | --- |
| **Species** | **Colony location** | **Data collection years*** | **Study method** | **Dual foraging?** | **Citation** | |
| Short-tailed shearwater | Althorpe Island, South Australia | 2005 – 2007 | nest attendance, chick feeding rate | Y | (Einoder et al. 2013) | |
|  |  | 2005-2007 | satellite transmitters, chick weighing | Y | (Einoder et al. 2011) | |
|  | Montague Island, New South Wales, Australia | 1997 | nest attendance, adult and chick weighing | Y | (Schultz and Klomp 2000) | |
|  | Bruny Island, Tasmania | 1997 | nest attendance | Y | (Weimerskirch and Cherel 1998) | |
|  | Wedge Island, Southern Tasmania | 2008 | GLS, TDR | Y | (Vertigan 2010) | |
| Shy albatross | Albatross Island, western Bass Strait | 1995-1998 | nest attendance, chick weighing | N | (Hedd et al. 2002) | |
| Sooty shearwater | Kidney Island, Falkland islands | 2008-2010 | GLS | N | (Hedd et al. 2014) | |
|  | Snares Islands, New Zealand | 2003 | GLS | Y | (Shaffer et al. 2009) | |
|  | Codfish Island, New Zealand | 2005 | GLS | Y | (Shaffer et al. 2009) | |
|  | Mana Island, New Zealand | 2005 | GLS | Y | (Shaffer et al. 2009) | |
|  | North-east Island, Snares Island, New Zealand | 1995 | nest attendance, chick weighing | Y | (Weimerskirch 1998) | |
| Streaked shearwater | Nago area, Mikura Island, Japan | 2003, 2006 | TDR, stomach contents | Y | (Matsumoto et al. 2012) | |
|  |  | 2005 | nest attendance, automatic weighing | N | (Ochi et al. 2010) | |
|  | Mikura island, Japan | 2003-2006 | nest attendance, weight | Y | (Ochi et al. 2016) | |
|  | Awashima Island, Japan | 2011-2013 | GPS | Y^ß^ | (Matsumoto et al. 2017) | |
|  | Sangan Island, Japan | 2005 | nest attendance, weight | Y | (Ochi et al. 2016) | |
| Thick-billed murre | Coats Island, Nunavut, Canada | 2000 – 2007 | TDR, prey inspection | N | (Elliott et al. 2009a) | |
| Thin-billed prion | Mayes Island, Kerguelen Islands, Southern Indian Ocean | 1993 | chick weighing | Y | (Weimerskirch et al. 1995) | |
|  |  | 1997 | chick weighing | Y | (Duriez et al. 2000) | |
|  | Kerguelen Island, Southern Indian Ocean | 1992 | nest attendance, adult weighing | Y | (Weimerskirch et al. 1994) | |

| **Table S1.** Data extracted from studies included in the review (continued) | | | | | |  |
| --- | --- | --- | --- | --- | --- | --- |
| **Species** | **Colony location** | **Data collection years*** | **Study method** | **Dual foraging?** | **Citation** | |
| Wandering albatross | Bird Island, South Georgia | 1996-1997, 2002, 2004 | radio transmitter, GPS | N | (Phillips et al. 2009) | |
|  |  | 1997 | nest attendance, chick weighing | Y | (Berrow and Croxall 2001) | |
|  |  | 2009 | GPS, salt water immersion, stomach temperature probe | Y | (Pereira et al. 2018) | |
|  | Crozet island, southern Indian Ocean | 1990 -1992 | nest attendance, adult weighing | Y | (Weimerskirch et al. 1994) | |
|  | Kerguelen Island, Southern Indian Ocean | 1990 -1992 | nest attendance, adult weighing | Y | (Weimerskirch et al. 1994) | |
|  | Possession Island, Crozet Islands, southern Indian Ocean | 1990 | ARGOS transmitters | Y | (Weimerskirch et al. 1993) | |
|  |  | 1992 | nest attendance | Y | (Weimerskirch et al. 1997) | |
|  | Marion Island, subantarctic Indian Ocean | 1996-1997 | PTT, GLS, nest attendance, diet | Y^ß^ | (Nel et al. 2002) | |
| Waved albatross | Isla Espanola, Galapagos | 1996 | PTT | Y | (Fernandez et al. 2001) | |
| Wedge-tailed shearwater | Heron Island, Australia | 2001 | nest attendance, weighing of chick and adults | Y | (Congdon et al. 2005) | |
|  |  | 2003 | nest attendance, chick weighing | Y | (Peck and Congdon 2005) | |
|  |  | 2006, 2011, 2012 | PTT, nest attendance | Y | (McDuie et al. 2015) | |
|  |  | 2015 | nest attendance, chick weighing, isotope, GPS | Y | (Miller 2018) | |
|  | Lord Howe Island, southwest Pacific Ocean | 2004 | nest attendance, chick weighing | N | (Peck and Congdon 2005) | |
|  |  | 2015 | nest attendance, chick weighing, isotope, GPS | Y | (Miller 2018) | |
|  |  | 2016 | nest attendance, chick weighing, isotope, GPS | N | (Miller 2018) | |
|  | Grand Anse, Reunion Island, Indian Ocean | 2016-2017 | GPS | Y | (Keys 2018) | |
|  | Tern Island, Hawaii | 1996-1997 | nest attendance, weighing adults and chicks | N | (Baduini 2002) | |

| **Table S1.** Data extracted from studies included in the review (continued) | | | | | |  |
| --- | --- | --- | --- | --- | --- | --- |
| **Species** | **Colony location** | **Data collection years*** | **Study method** | **Dual foraging?** | **Citation** | |
| Westland petrel | Punakaiki, Westland, New Zealand | 2012, 2015, 2016 | I-got-u loggers | N | (Waugh et al. 2018) | |
|  |  | 1995-1996 | PTT, diet | Y | (Freeman et al. 2001) | |
| White-chinned petrels | Possession Island, Crozet Islands, southern Indian Ocean | 1997 | nest attendance, satellite tracking | Y | (Catard et al. 2000) | |
| Yellow-nosed albatross | Amsterdam islands, southwestern Indian Ocean | 1991-1992 | weighing adult, monitor return | Y | (Weimerskirch et al. 1994) | |
|  | Pointe d’Entrecasteaux, Amsterdam Island, southern Indian Ocean | 1995-1996 | nest attendance | N | (Weimerskirch et al. 2000) | |
|  |  | 1996 | PTT, nest attendance, diet | N | (Pinaud et al. 2005) | |
|  |  | 2001 | PTT, nest attendance, diet | Y | (Pinaud et al. 2005) | |
| GPS, Global Postitioning System; TDR, Time Depth Recorder; PTT, Platform Transmitter Terminal; GLS, Global Location Sensor; GPS-GSM, GPS-Global System for Mobile Communications loggers  *Any year where data were collected during chick-rearing  ^ʎ^ This data is also reported by Nel in a PhD thesis (2002).  ^Ʈ^ A subset of this data, the TDR data, is also reported in Brown and colleagues' paper (2012).  ^ǂ^ A subset of this data collected in Magdalenefjorden in 2009 was first reported by Jakubas and colleagues (2012).  ^ß^ Only males performed the dual foraging strategy. | | | | | | |
